# The Enigmatic Thelebolaceae (Thelebolales, Leotiomycetes): One New Genus *Solomyces* and Five New Species

**DOI:** 10.3389/fmicb.2020.572596

**Published:** 2020-10-29

**Authors:** Zhiyuan Zhang, Chunbo Dong, Wanhao Chen, Quanrong Mou, Xiaoxiao Lu, Yanfeng Han, Jianzhong Huang, Zongqi Liang

**Affiliations:** ^1^Institute of Fungus Resources, Department of Ecology, College of Life Sciences, Guizhou University, Guiyang, China; ^2^Department of Microbiology, Guiyang College of Traditional Chinese Medicine, Guiyang, China; ^3^Key Laboratory of Plant Resource Conservation and Germplasm Innovation in Mountainous Region (Ministry of Education), Guizhou University, Guiyang, China; ^4^Engineering Research Center of Industrial Microbiology, Ministry of Education, Fujian Normal University, Fuzhou, China

**Keywords:** taxonomy, phylogeny, Thelebolales, new genus, new species

## Abstract

The family Thelebolaceae belongs to the order Thelebolales, class Leotiomycetes, and contains 22 genera. In this study, we introduce a new genus *Solomyces* gen. nov. in the family Thelebolaceae, which is supported by morphological observation and multilocus-based [internal transcribed spacers (ITS) + *LSU* and ITS + *LSU*+ *MCM7*+ *EF1A*+ *RPB2*] phylogenetic analysis. Maximum-likelihood and Bayesian phylogenetic inference analyses indicated that *Solomyces* is a distinct genus within this family. The new genus is compared against related Thelebolaceae genera, and its description and illustration are provided. This genus comprises one new species and one unnamed species (including two strains). We also report the addition of four new species – *Pseudogymnoascus shaanxiensis*, *Pseudogymnoascus guizhouensis*, *Pseudogymnoascus sinensis*, and *Geomyces obovatus* – in the family Thelebolaceae and present their morphological and phylogenetic characterizations.

## Introduction

The class Leotiomycetes (Pezizomycotina) was erected by Eriksson and Winka (1997) to accommodate inoperculate discomycetes. Fungi in the class Leotiomycetes are ecologically diverse and include mycorrhizas, root and leaf endophytes, plant pathogens, aquatic and aeroaquatic hyphomycetes, mammalian pathogens, and saprobes (Johnston et al., 2019). Leotiomycetes currently comprises 19 orders and order-level clades, 54 families, and 13 family level clades (Ekanayaka et al., 2019; Karunarathna et al., 2020). Previously, however, this class included a wide range of taxa based on traditional morphological taxonomy (Korf, 1973; Spooner, 1987), and the current classification of Leotiomycetes is still largely based on morphologically defined taxa, especially at higher taxonomic levels (Johnston et al., 2019). Nevertheless, sexual or asexual morphs of many Leotiomycetes taxa are not recorded, and a few links between sexual and asexual morphs in Leotiomycetes have been confirmed (Sutton and Hennebert, 1995; Sati and Pathak, 2016; Ekanayaka et al., 2017; Johnston et al., 2019). Thelebolales, an order in the class Leotiomycetes, consists of Thelebolaceae and the *Alatospora*–*Miniancora* clade, in which some genera (e.g., *Caccobius*, *Coprobolus*, and *Leptokalpion*) are erected based on their sexual, but not asexual, morphology (Wijayawardene et al., 2017).

Haeckel (1894) introduced the order Thelebolales; however, the taxonomy of this order has long been contentious. Some researchers believed that the order contained only one family (De Hoog et al., 2005), whereas others suggested that at least two families were involved (Ekanayaka et al., 2019; Johnston et al., 2019; Batista et al., 2020). The family Thelebolaceae is important in the order Thelebolales because of several species that can produce antifreeze proteins, ice-binding proteins, and some secondary metabolites with potential application values that offer valuable resources for biotechnological exploitation (Batista et al., 2020); the family was introduced by Eckblad (1968) and typified by the genus *Thelebolus* with *Thelebolus stercoreus* as the type species. Members of this family are characterized by absent, apothecial, or cleistothecial ascomata (Van Brummelen, 1985; Stchigel et al., 2001; De Menezes et al., 2017; Ekanayaka et al., 2019). During an extensive diversity survey of *Geomyces* and allied genera in China, we gathered a collection of fungal isolations. In this study, we introduced their morphological, cultural, and phylogenetic characterization and propose one new genus and five new species.

## Materials and Methods

### Isolates and Morphology

Soil samples were obtained from Dali City, Yunnan Province; Guiyang City, Guizhou Province; Xi’an and Hanzhong City, Shaanxi Province; and Yichang City, Hubei Province, China. Samples were treated according to the method described by Zhang et al. (2019). Fungi were isolated using a modified baiting technique with chicken feathers as the substrate (Vanbreuseghem, 1952). The feathers were washed, thoroughly rinsed with distilled water, dried, cut into 2-cm fragments, and autoclaved. Plates containing soil material and sterile feathers were incubated at room temperature for 1 month. Fungi were isolated and purified using a conventional dilution technique described by Zhang et al. (2019), as follows: 2 g of soil sample was suspended in 9 ml of distilled water, and the prepared suspension was vortexed, diluted to 1:10,000, and cultured on Sabouraud’s dextrose agar (SDA; 10 g of peptone, 40 g of dextrose, 20 g of agar, 1 L of ddH_2_O) supplemented with chloramphenicol and cycloheximide. The plates were incubated at 25°C until fungal colony growth was observed. The axenic strains were then transferred to potato dextrose agar (PDA; Shanghai Bio-way Technology Co., Ltd., China) plates for purification. Dried holotypes were deposited in the Mycological Herbarium of the Institute of Microbiology, Chinese Academy of Sciences, Beijing, China (HMAS), or the Institute of Fungus Resources, Guizhou University (GZUIFR, formally the Herbarium of Guizhou Agricultural College; code, GZAC). Ex-type strains and other strains were deposited in the China General Microbiological Culture Collection Center (CGMCC) or the GZUIFR.

The pure strains were incubated on PDA at 25°C for 14 days in the dark to determine the macroscopic characteristics, diameters, and colony colors (surface and reverse). The characterization and measurement of fungal microscopic characteristics were performed in 25% lactic acid. Images were obtained using an optical microscope (OM, DM4 B, Leica, Germany) with differential interference contrast (DIC). The taxonomic descriptions and names of the new taxa were introduced into MycoBank^1^ and Faces of Fungi^2^.

### DNA Extraction, PCR Amplification, and Sequencing

Total DNA was extracted using the BioTeke Fungus Genomic DNA Extraction kit (DP2032, BioTeke, Beijing, China) following the manufacturer’s instructions. Total DNA was used for the amplification and sequencing of five fragments: ribosomal internal transcribed spacers (ITS; primers ITS1/ITS4) (White et al., 1990), the 28S LSU rRNA gene (primers LROR/LR7) (Vilgalys and Hester, 1990; Vilgalys and Sun, 1999), translation elongation factor 1 alpha (*EF1A*; primers 983F/2218R) (Rehner and Buckley, 2005), RNA polymerase II subunit 2 (*RPB2*; primers fRPB2-7cF/RPB2-3053b) (Liu et al., 1999; Reeb et al., 2004), and minichromosomal maintenance protein 7 (*MCM7*; primers MCM7-709/MCM7-1348) (Schoch et al., 2009). The PCR products were sequenced with the abovementioned primers at a commercial sequencing service provider (Shanghai Sangon Biological Engineering Technology & Services Co., Shanghai, China) in an ABI 3730xl DNA Analyzer, using the Sanger method. The consensus sequences were obtained using the SeqMan software v. 7 (DNASTAR Lasergene, Madison, WI, United States). Sequences generated in this study were deposited in GenBank (Table 1).

**TABLE 1:**
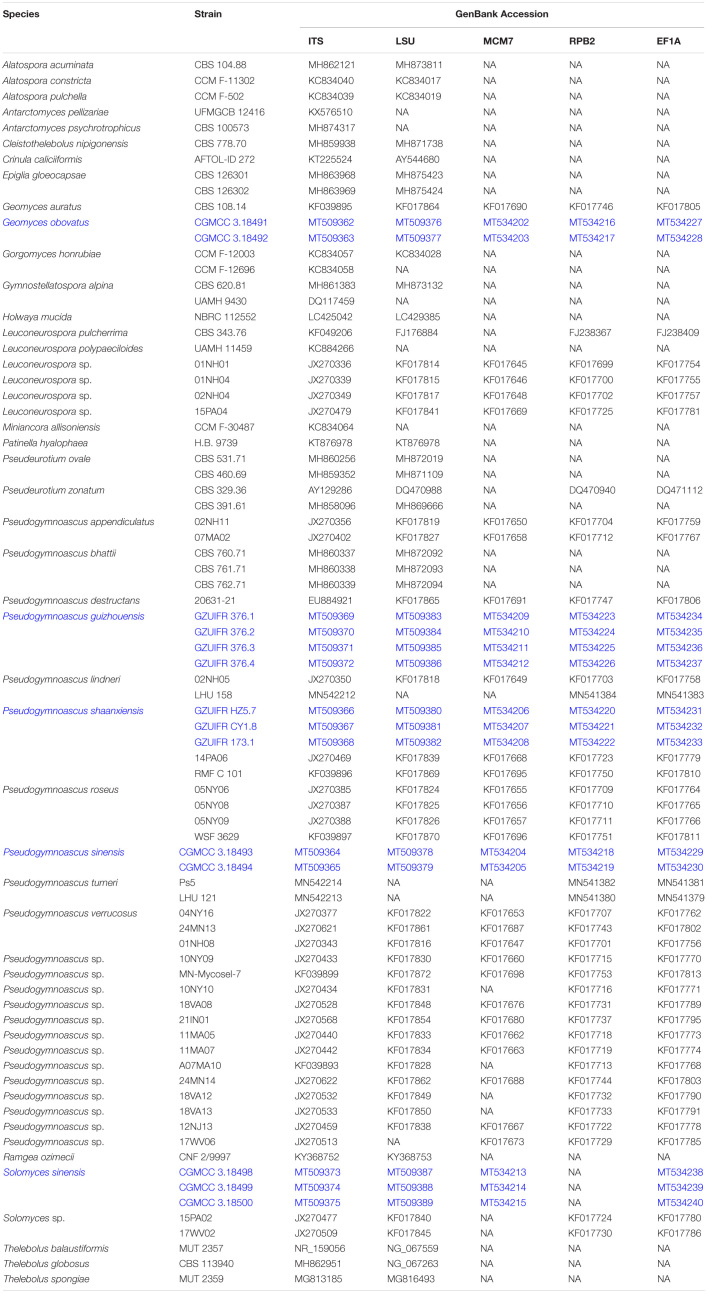
List of strains used in the phylogenetic analysis.

*CBS, Westerdijk Fungal Biodiversity Institute, Utrecht, Netherlands; CGMCC, China General Microbiological Culture Collection Center, Beijing, China; GZUIFR, Guizhou University, Institute of Fungus Resources; CCM, Czech Collection of Microorganisms, Brno, Czechia; NBRC (IFO), Institute for Fermentation, Osaka, Japan; UAMH, UAMH Centre for Global Microfungal Biodiversity, Toronto, ON, Canada; MUT, Mycotheca Universitatis Taurinensis, Turin, Italy; NA, not available. DNA sequences for the new isolates were in blue.*

### Phylogenetic Analyses

Sequence data were selected from data previously published by Crous et al. (2019), Ekanayaka et al. (2019), Johnston et al. (2019), and Minnis and Lindner (2013) and then downloaded from GenBank for molecular phylogenetic analyses (Table 1). Multiple sequence alignments for ITS, *LSU*, *MCM7*, *RPB2*, and *EF1A* were carried out using MAFFT v7.037b (Katoh and Standley, 2013). Sequence editing was performed with MEGA6 (Tamura et al., 2013). The concatenated ITS and *LSU*, and ITS, *LSU*, *MCM7*, *RPB2*, and *EF1A* sequences were assembled by SequenceMatrix v.1.7.8 (Vaidya et al., 2011). Gene concordance was assessed with the “hompart” command in PAUP4.0b10 (Swofford, 2002).

Sequence alignment and maximum-likelihood (ML) and Bayesian inference (BI) phylogenetic analyses were performed according to the methodology described by Zhang et al. (2019). Maximum-likelihood (ML) analyses were constructed with IQ-TREE v. 1.6.11 (Nguyen et al., 2015). The best-fit model of substitution for each locus was estimated using IQ-TREE’s ModelFinder function (Kalyaanamoorthy et al., 2017) under the Bayesian information criterion (BIC). Bootstrap analysis was performed using the ultrafast bootstrap approximation (Minh et al., 2013) with 1,000 replicates, and bootstrap support (BS) ≥90% was considered as statistically significant. For Bayesian inference (BI), a Markov chain Monte Carlo (MCMC) algorithm was used to generate phylogenetic trees with Bayesian probabilities using MrBayes v.3.2 (Ronquist et al., 2012) for the combined sequence datasets. The selection of the best-fit nucleotide substitution model for each locus was calculated by the Akaike information criterion (AIC) with ModelTest v.3.7 (Posada and Crandall, 1998). After the BI analyses, both runs were examined with Tracer v.1.5 (Drummond and Rambaut, 2007) to determine burn-in and check for convergence.

Two multilocus phylogenies were analyzed to evaluate the various generic placements and establish the phylogenetic relationships in Thelebolaceae. The genus placement was evaluated based on a concatenated ITS and *LSU* dataset. This phylogeny was constructed to assess if *Solomyces* is a well-delimited genus. The second multilocus phylogeny was based on a concatenated alignment of ITS, *LSU*, *MCM7*, *RPB2*, and *EF1A* sequences and included 49 isolates of *Geomyces* and its allied genera. This analysis was performed to evaluate the generic boundaries and species groupings within the genera *Solomyces*, *Geomyces*, and *Pseudogymnoascus*. All the trees were displayed in FigTree 1.4.2^3^ and edited in Microsoft Paint. DNA alignments have been deposited at TreeBASE.

## Results

### Phylogenetic Analyses

**FIGURE 1:**
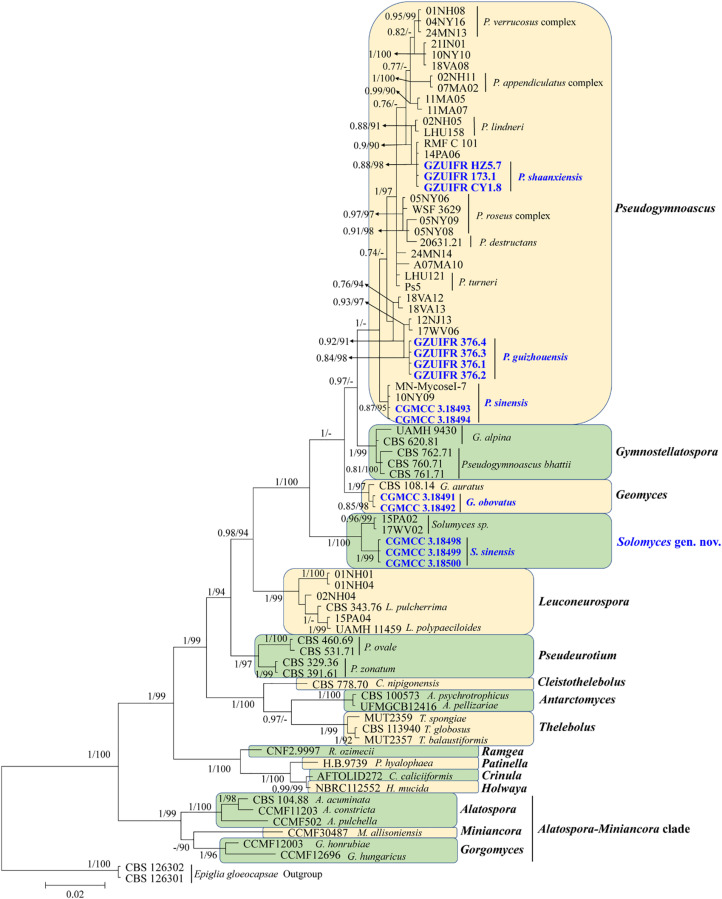
Phylogenetic relationships among the genera in Thelebolales based on Bayesian inference and maximum-likelihood analyses of the concatenated internal transcribed spacers (ITS) and *LSU* dataset. Posterior probabilities ≥0.7 and maximum-likelihood bootstrap values ≥90% are shown above the internal branches. The new isolates are presented in bold and blue.

**FIGURE 2:**
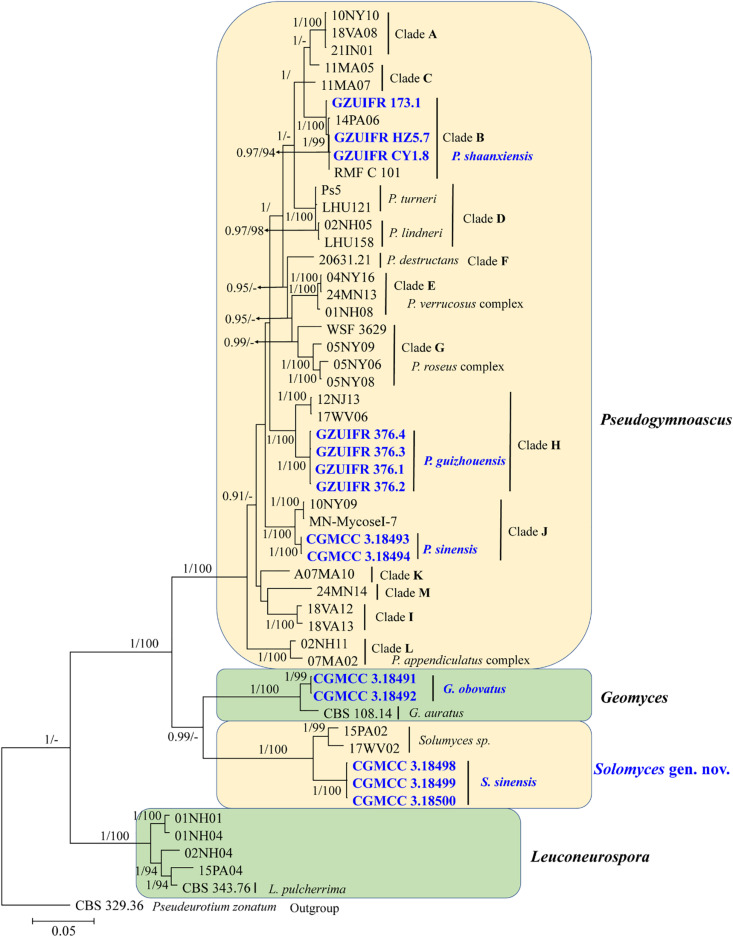
The consensus tree from the Bayesian inference and *Geomyces* and its allied genera based on the ITS, *LSU*, *MCM7*, *RPB2*, and *EF1A* concatenated dataset. Posterior probabilities ≥0.7 and maximum-likelihood bootstrap values ≥90% are shown above the internal branches. The new isolates are presented in bold and blue.

The first concatenated matrix contains 1,220 nucleotides, i.e., 445 from the ITS and 775 from the *LSU*. The second concatenated alignment contained 2,808 nucleotides (ITS: 434, *LSU*: 806, *MCM7*: 503, *RPB2*: 437, and *EF1A*: 628). The best-fit evolutionary model for each locus in the two datasets is listed in Table 2. The tree topology of the Bayesian inference agrees with that of the ML tree (Figures 1, 2). The phylogenies indicated that each genus clusters into a monophyletic clade (Figure 1). In the phylogenetic tree, the new genus *Solomyces* forms a well-supported (1 BYPP/100 MLBS) clade separated from other genera in Thelebolaceae (Figure 1). *Solomyces sinensis* represents a separate subclade and is located within the new genus near another unnamed species (includes two strains, 15PA02 and 17WV02, from Hibernacular soil). Furthermore, all the new species of *Pseudogymnoascus* and *Geomyces* are placed in a distinct branch.

**TABLE 2:** The best-fit evolutionary model in the phylogenetic analyses.

Dataset/phylogenetic analysis		Model				
		ITS	LSU	MCM7	RPB2	EF1A
First dataset	ML analysis	TIM2e + I + G4	K2P + R2			
	BI analysis	GTR + I + G	GTR + I + G			
Second dataset	ML analysis	TIM2e + R3	TPM2u + F + I	TNe + I + G4	TNe + I + G4	TIM2e + R3
	BI analysis	SYM + I + G	GTR + I	HKY + I + G	K80 + I + G	SYM + I + G

*ML, maximum likelihood; BI, Bayesian inference.*

### Taxonomy

In this section, *Solomyces* Zhi.Y. Zhang, Y.F. Han and Z.Q. Liang, gen. nov. and five new species – *S. sinensis* Zhi.Y. Zhang, Y.F. Han and Z.Q. Liang, sp. nov.; *Geomyces obovatus* Zhi.Y. Zhang, Y.F. Han and Z.Q. Liang, sp. nov.; *Pseudogymnoascus guizhouensis* Zhi.Y. Zhang, Y.F. Han and Z.Q. Liang, sp. nov.; *Pseudogymnoascus shaanxiensis* Zhi.Y. Zhang, Y.F. Han and Z.Q. Liang, sp. nov.; and *Pseudogymnoascus sinensis* Zhi.Y. Zhang, Y.F. Han and Z.Q. Liang, sp. nov. – are described and illustrated. We also propose a new combination, *Gymnostellatospora bhattii* (Samson) Zhi.Y. Zhang, Y.F. Han and Z.Q. Liang, comb. nov. based on phylogenetic analyses.

#### *Geomyces obovatus* Zhi.Y. Zhang, Y.F. Han **and** Z.Q. Liang, sp. nov.

**FIGURE 3:**
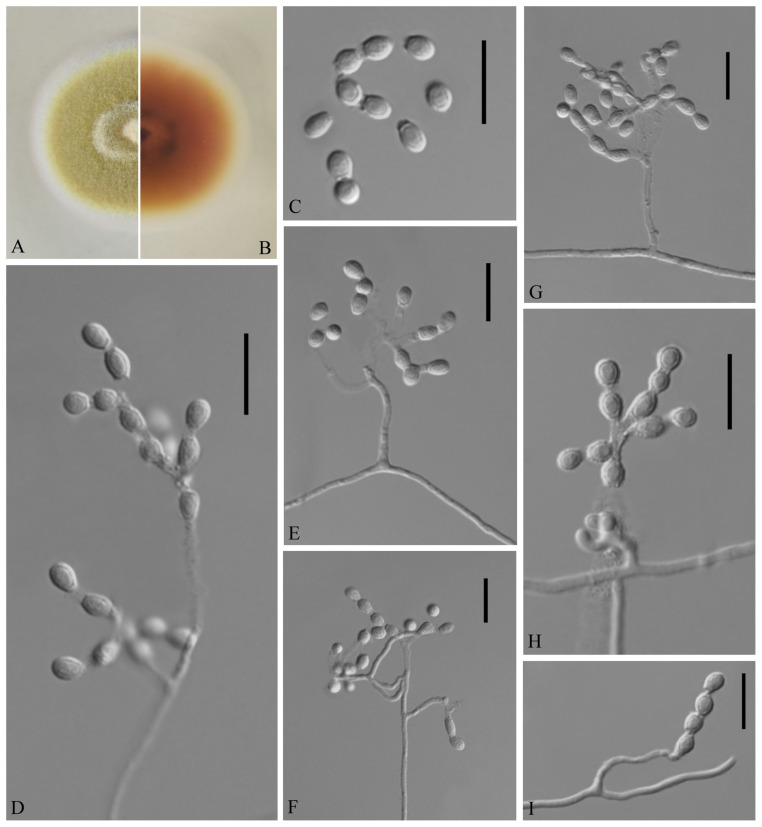
*Geomyces obovatus* [from ex-holotype China General Microbiological Culture Collection Center (CGMCC) 3.18491]. **(A,B)** The front and reverse of a *G. obovatus* colony on potato dextrose agar (PDA) after 14 days at 25°C. **(C–I)** Conidiophores and conidia. Scale bars = 10 μm.

MycoBank number: MB 835718, Facesoffungi number: FoF 08691 (Figure 3).

Etymology: Referring to the obovoid conidia.

Holotype: permanently preserved in a metabolically inactive state, GZAC 20.8.

Description based on GZAC 20.8. Asexual: Colonies on PDA, reaching 28–30 mm in diameter after 14 days at 25°C, slightly felty to floccose, margin identified, khaki at center, and white at edge; reverse hazel. Aerial mycelium abundant, smooth and thin walled, septate, 0.5- to 2-μm wide. Racquet hyphae absent. Conidia abundant, normally terminal and intercalary conidia in a series of 1–4, rarely solitary on short stalk, smooth or echinulate, 3.0–5.0 × 2.5–4.0 μm (*av*. = 3.5 × 2.5 μm, *n* = 50). Terminal conidia obovoid, rarely subglobose; intercalary conidia alternate and pyriform, barrel or irregularly shaped.

Sexual morph: not observed.

Geographical distribution: China.

Material examined: China, Guizhou, Guiyang, Qianlingshan Park, 26°60′N, 106°69′E, from soil beside a road, September 2016, Zhi. Y. Zhang (GZAC 20.8 – holotype; CGMCC 3.18491 = GZUIFR QL20.8.1 – ex-type living cultures; ibid., CGMCC 3.18492 = GZUIFR QL20.8.2). The living cultures were kept in sterile 30% glycerol and deposited in a −80°C freezer.

Notes: The *Geomyces* was introduced by Traaen (1914), and several species have subsequently been described. Currently, the genus contains seven species (Traaen, 1914; Dal Vesco, 1957; Sigler and Carmichael, 1976; Hocking and Pitt, 1988; Li and Cui, 1989; Luo et al., 2016; Chen et al., 2017): *Geomyces auratus*, *Geomyces asperulatus*, *Geomyces vinaceus*, *Geomyces pulvereus*, *Geomyces laevis*, *Geomyces guiyangensis*, and *Geomyces fujianensis*. *G. asperulatus* and *G*. *vinaceus* likely belong to *Pseudogymnoascus* (Minnis and Lindner, 2013); *G*. *pulvereus* and *G*. *laevis* do not have any available sequence data; *G*. *pulvereus* lacks intercalary conidia (Hocking and Pitt, 1988), and *G*. *laevis* has oblong-elliptical or barrel-shaped intercalary conidia (Li and Cui, 1989). Only ITS sequences are available for *G*. *guiyangensis* and *G*. *fujianensis*, and the ITS data for CGMCC 3.18491 (the type strain of *G. obovatus*) show similarity to those of *G*. *guiyangensis* (strain G014512) (465/509; 91%, with 4 gaps) and *G*. *fujianensis* (strain G242) (464/526; 88%, with 11 gaps); however, the latter two species have no intercalary conidia (Luo et al., 2016; Chen et al., 2017). The proposed new species, *G*. *obovatus*, is morphologically and phylogenetically related to *G. auratus*, but they can be distinguished by their ITS (512/524; 97%, with three gaps), *LSU* (931/938; 99%, no gaps), *MCM7* (592/619; 95%, no gaps), *RPB2* (696/717; 97%, no gaps), and *EF1A* (843/855; 98%, with one gap) sequence data.

#### *Gymnostellatospora bhattii* (Samson) Zhi.Y. Zhang, Y.F. Han and Z.Q. Liang, comb. nov.

Basionym: *Pseudogymnoascus bhattii* Samson, *Acta Botanica Neerlandica* 21: 519. 1972.

Notes: This species was erected by Samson (1972) based on morphological characteristics. However, our phylogenetic analysis based on Thelebolaceae ITS and *LSU* data indicated that this species was closely related to *Gymnostellatospora* and separated from *Pseudogymnoascus*. Based on morphological characteristics, it was difficult to distinguish the closely related species, and even the genera, through traditional taxonomy, and modern phylogenetic methods were a very important adjunct. We therefore transferred *P*. *bhattii* to *Gymnostellatospora* and named the species *G. bhattii*.

#### *Pseudogymnoascus guizhouensis* Zhi.Y. Zhang, Y.F. Han and Z.Q. Liang, sp. nov.

**FIGURE 4:**
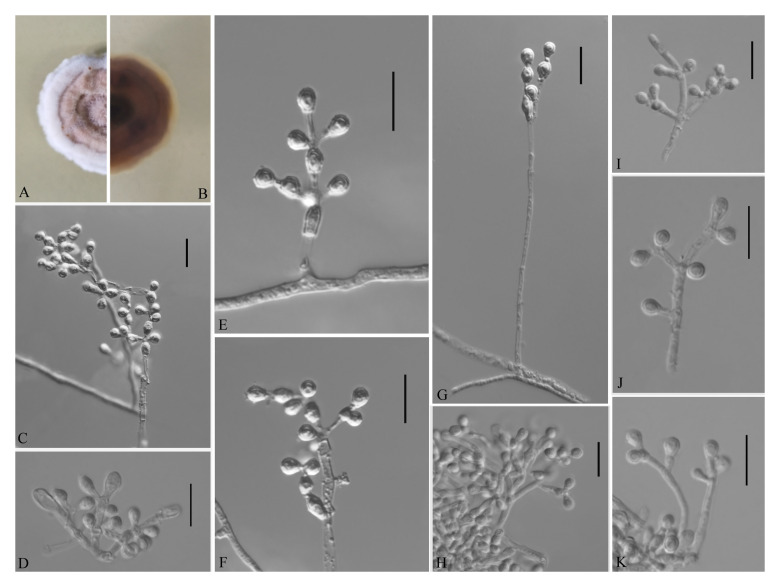
*Pseudogymnoascus guizhouensis* [from ex-holotype Institute of Fungus Resources, Guizhou University (GZUIFR) 376.1]. **(A,B)** The front and reverse of a *P. guizhouensis* colony on PDA after 14 days at 25°C. **(C–K)** Conidiophores and conidia. Scale bars = 10 μm.

MycoBank number: MB 835716, Facesoffungi number: FoF 08692 (Figure 4).

Etymology: Refers to the region from which the fungus was isolated.

Holotype: permanently preserved in a metabolically inactive state, GZAC 376.

Description based on GZAC 376. Asexual: Colonies on PDA, reaching 20–23 mm in diameter after 14 days at 25°C, elevate, powdery, floccose, margin identified, locally indented, pale purple at center and white at the edge, producing a clear exudate; reverse brown. Aerial mycelium abundant, smooth and thin walled, septate, 1.5- to 3.0-μm wide. Racquet hyphae absent. Conidia normally borne on verticillate branches, terminal, intergrading with intercalary conidia, sometimes borne laterally and solitary on hyphae, smooth walled or echinulate, obovoid, pyriform, subglobose or clavate, 3.0–5.5 × 3.0–3.5 μm (*av*. = 4.0 × 3.0 μm, *n* = 50). Intercalary conidia are borne on the outer branches of the hyphae or verticillate hyphae, alternate, in series of 1–2, smooth walled or echinulate, cuneiform, barrel shaped, 3.5–6.0 × 3.0–3.5 μm (*av*. = 4.0 × 3.0 μm, *n* = 50).

Sexual morph: not observed.

Geographical distribution: China.

Material examined: China, Guizhou, Guiyang, Guizhou University, 26°45′N, 106°67′E, from epiphytic soil of *Cinnamomum camphora*, June 2019, Zhi. Y. Zhang (GZAC 376 – holotype; GZUIFR 376.1 – ex-type living culture; ibid., GZUIFR 376.2; ibid., GZUIFR 376.3; ibid., GZUIFR 376.4). The living cultures were kept in sterile 30% glycerol and deposited in a −80°C freezer.

Notes: Morphologically, *Pseudogymnoascus guizhouensis* is similar to *Pseudogymnoascus linderi*, *Pseudogymnoascus turneri* (both isolated from sediment), and *Pseudogymnoascus destructans* (isolated from a wing of a small brown bat, *Myotis lucifugus*), based on the obovoid and subglobose conidia. However, *P. guizhouensis* differs from *P. linderi* and *P. turneri* as it has cuneiform, barrel-shaped intercalary conidia (the intercalary conidia of *P. linderi* and *P. turneri* are globose to truncate) (Crous et al., 2019). *P. guizhouensis* can be distinguished from *P. destructans* by the size of conidia and intercalary conidia (4.0 × 3.0 μm vs. 5.0–12.0 × 2.0–3.5 μm, respectively) (Gargas et al., 2009). Phylogenetically, our isolates GZUIFR 376.1, GZUIFR 376.2, GZUIFR 376.3, and GZUIFR 376.4 cluster together very well and form a single clade separated from other *Pseudogymnoascus* species (Figures 1, 2).

#### *Pseudogymnoascus shaanxiensis* Zhi.Y. Zhang, Y.F. Han and Z.Q. Liang, sp. nov.

**FIGURE 5:**
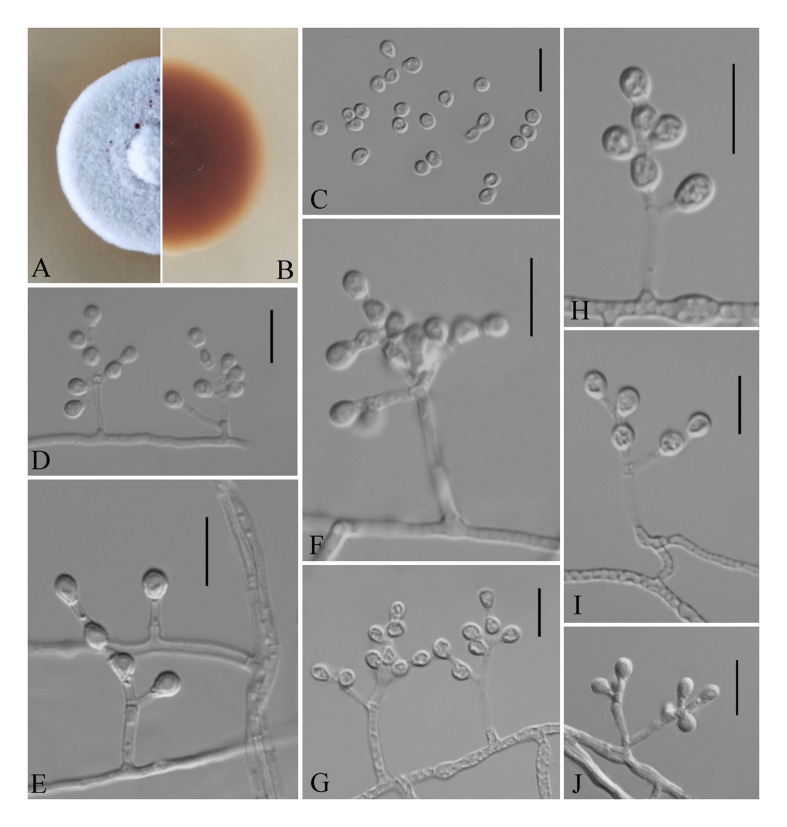
*Pseudogymnoascus shaanxiensis* (from ex-holotype GZUIFR 173.1). **(A,B)** The front and reverse of a *P. shaanxiensis* colony on PDA after 14 days at 25°C. **(C)** Conidia. **(D–J)** Conidiophores and conidia. Scale bars = 10 μm.

MycoBank number: MB 835715, Facesoffungi number: FoF 08689 (Figure 5).

Etymology: Refers to Shaanxi, the province where the isolate was collected.

Holotype: permanently preserved in a metabolically inactive state, HMAS 255395.

Description based on HMAS 255395. Asexual: Colonies on PDA, reaching 21–23 mm in diameter after 14 days at 25°C, velvety to floccose, margins regular, white, producing a diffusible faint yellow pigment and clear exudates; reverse brown. Hyphae hyaline, smooth-walled, septate, 1.5- to 2.5-μm wide. Racquet hyphae absent. Conidia abundant, pyriform, sometimes subglobose, smooth 3.5–5.0 × 2.5–3.0 μm (*av*. = 4.0 × 3.0 μm, *n* = 50). Intercalary conidia subglobose, pyriform, or irregularly shaped, smooth, 3.5–4.0 × 3.0–4.0 μm (*av*. = 3.5 × 3.0 μm, *n* = 50). Terminal and lateral conidia on hyphae, short stalk or side branch, solitary, always forming verticillate and opposite branches with an acute angle to the axis near the apex.

Sexual morph: not observed.

Geographical distribution: China and America.

Material examined: China, Shaanxi, Xi’an, Xincheng district, 34°27′N, 108°95′E, from epiphytic soil of *Broussonetia papyrifera*, September 2018, Zhi. Y. Zhang (HMAS 255395 – holotype; GZUIFR 173.1 – ex-type living culture); Hanzhong, Shaanxi province, from epiphytic soil of *Trachycarpus fortunei*, September 2018, Zhi. Y. Zhang (GZUIFR HZ5.7); Yichang, Hubei province, from soil beside a park, September 2018, Zhi. Y. Zhang (GZUIFR CY 1.8). The living cultures were kept in sterile 30% glycerol and deposited in a −80°C freezer.

Notes: Morphologically, *P. shaanxiensis* resembles *Pseudogymnoascus appendiculatus* and *Pseudogymnoascus verrucosus* because of the pyriform conidia. However, *P. shaanxiensis* differs from *P. appendiculatus* and *P. verrucosus* based on its subglobose, pyriform intercalary conidia (the intercalary conidia of *P. appendiculatus* and *P. verrucosus* are subglobose to elongate) (Rice and Currah, 2006). Phylogenetically, our isolates GZUIFR 173.1, GZUIFR HZ5.7, and GZUIFR CY 1.8 cluster with 14PA06 and RMF C 101 in a distinct subclade and are separated from other clades (Figures 1, 2). Furthermore, since 2013 (Minnis and Lindner, 2013), isolates 14PA06 and RMF C 101 have remained an undescribed species owing to the lack of morphological characteristics. Therefore, we introduce *P. shaanxiensis* sp. nov. in this study.

#### ***Pseudogymnoascus sinensis*** Zhi.Y. Zhang, Y.F. Han **and** Z.Q. Liang, sp. nov.

**FIGURE 6:**
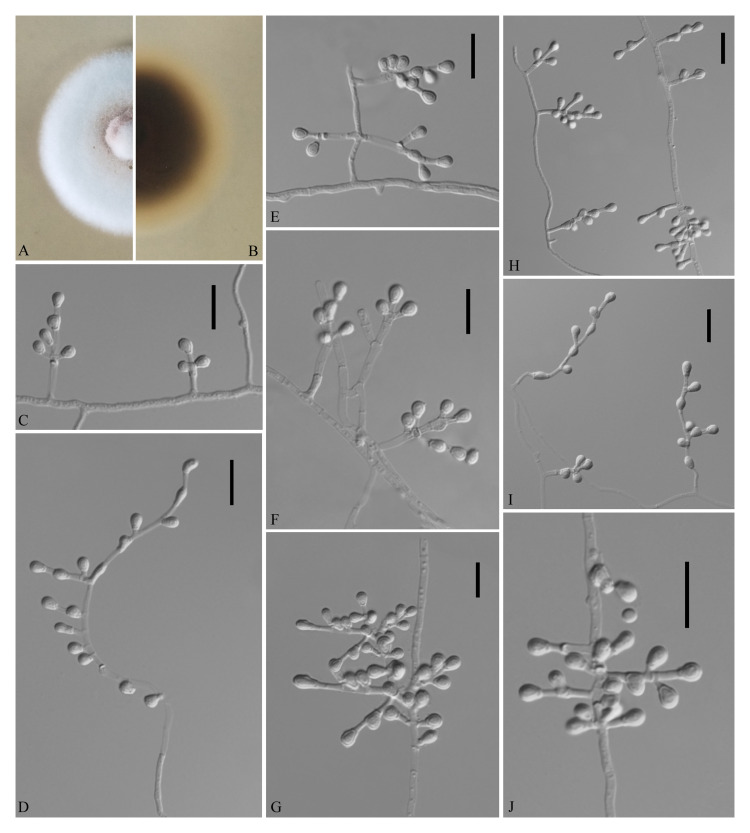
*Pseudogymnoascus sinensis* (from ex-holotype CGMCC 3.18493). **(A,B)** The front and reverse of a *P. sinensis* colony on PDA after 14 days at 25°C. **(C–J)** Conidiophores and conidia. Scale bars = 10 μm.

MycoBank number: MB 835717, Facesoffungi number: FoF 08690 (Figure 6).

Etymology: In reference to China, the country where the type specimen was obtained.

Holotype: permanently preserved in a metabolically inactive state, HMAS 255394.

Description based on HMAS 255394. Asexual: Colonies on PDA, reaching 20–21 mm in diameter after 14 days at 25°C, powdery, floccose, margin identified, light pink at the center and pewter at the edge; reverse brown. Aerial mycelium abundant, smooth and thin walled, septate, 1- to 2-μm wide. Racquet hyphae absent. Conidia abundant, terminal and lateral conidia on hyphae, short stalk or side branches, sometimes forming verticillate and opposite branches with an acute angle to the axis near the apex, solitary, obovoid, 3.0–5.0 × 2.5–3.0 μm (*av*. = 4.0 × 2.5 μm, *n* = 50). Intercalary conidia are borne on the outer branches of the hyphae or verticillate hyphae, smooth walled, drum, obovoid, pyriform, or irregularly shaped, 3.0–4.5 × 2.5–5.0 μm (*av*. = 3 × 3 μm, *n* = 50).

Sexual morph: not observed.

Geographical distribution: China.

Material examined: China, Guizhou, Guiyang, the Affiliated Hospital of Guizhou Medical University, 26°59′N, 106°71′E, from soil beside a road, September 2016, Zhi. Y. Zhang (HMAS 255394 – holotype; CGMCC 3.18493 = GZUIFR K278.1 – ex-type living cultures; ibid., CGMCC 3.18494 = GZUIFR K278.2). The living cultures were kept in sterile 30% glycerol and deposited in a −80°C freezer.

Notes: Morphologically, *P. sinensis* is similar to *P. linderi* and *P. turner*, based on its obovoid conidia. However, *P. sinensis* differs from *P. linderi* and *P. turneri* as it has drum, obovoid, pyriform, or irregularly shaped intercalary conidia (the intercalary conidia of *P. linderi* and *P. turneri* are globose to truncate) (Crous et al., 2019). Phylogenetically, our isolates CGMCC 3.18493 and CGMCC 3.18494 cluster together very well and form a single clade separated from other *Pseudogymnoascus* species (Figure 1).

#### *Solomyces* Zhi.Y. Zhang, Y.F. Han and Z.Q. Liang, gen. nov.

MycoBank number: MB 835713; Facesoffungi number: FoF 08688.

Etymology: *Solo*- (from *Solum*), in reference to its isolation from soil.

*Saprobic* on soil. Asexual morph: *Conidia*: terminal and lateral conidia borne on hyphae, short protrusions, or side branches. Conidia solitary, sometimes 2 in chains, pyriform, sometimes subglobose. Intercalary conidia abundant, olivary, subglobose to globose. Sexual morph: Unknown.

Geographical distribution: China and America.

Type species: *Solomyces sinensis* Zhi.Y. Zhang, Y.F. Han and Z.Q. Liang.

Notes: *Solomyces* is introduced to accommodate *Solomyces sinensis* and another unnamed species (containing two strains, 15PA02 and 17WV02, from Hibernacular soil in Pennsylvania and West Virginia, respectively; Minnis and Lindner, 2013). The morphology of *Solomyces* species is similar to that of *Geomyces* and the asexual morphs of *Pseudogymnoascus*. However, *Geomyces* differ in having terminal and lateral conidia borne on hyphae, short protrusions or side branches; intercalary conidia barrel shaped, and conidiophores abundant, always forming verticillate and opposite branches with an acute angle to the axis near the apex (Van Oorschot, 1980; Chen et al., 2017). As can be seen in Figures 1, 2, strains of these genera appear in distinct clades in a phylogeny based on multiple strains, thereby justifying the erection of the new genus *Solomyces*.

#### *Solomyces sinensis* Zhi.Y. Zhang, Y.F. Han and Z.Q. Liang, sp. nov.

**FIGURE 7:**
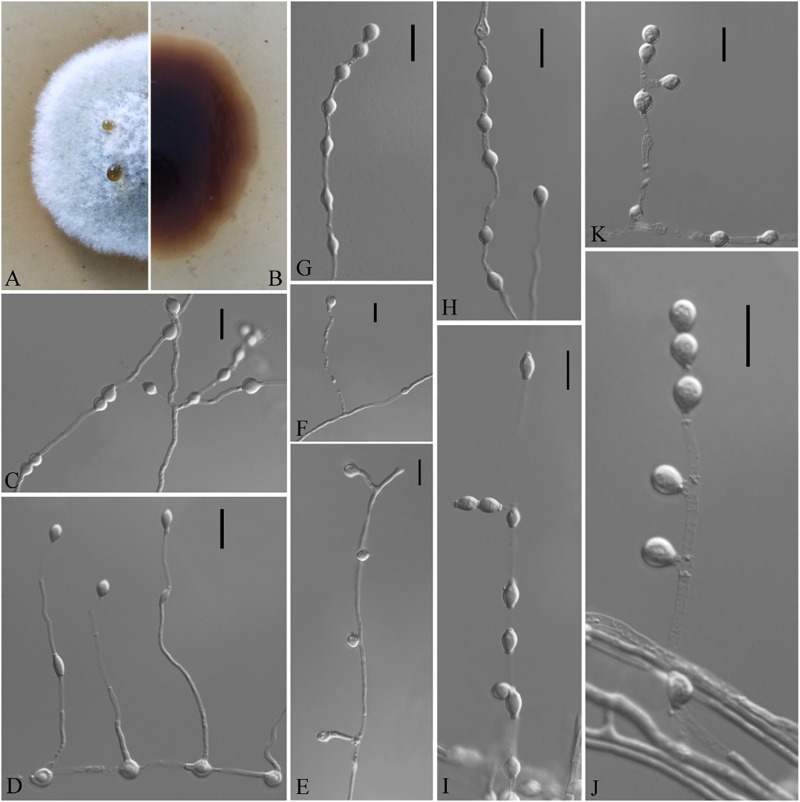
*Solomyces sinensis* (from ex-holotype CGMCC 3.18498). **(A,B)** The front and reverse of a *S. sinensis* colony on PDA after 14 days at 25°C. **(C–K)** Conidiophores and conidia. Scale bars = 10 μm.

MycoBank number: MB 835714, Facesoffungi number: FoF 08687 (Figure 7).

Etymology: In reference to China, the country where the type specimen was obtained.

Holotype: permanently preserved in a metabolically inactive state, HMAS 255397.

Description based on HMAS 255394. Asexual: Colonies on PDA, reaching 16–17 mm in diameter after 14 days at 25°C, elevate at the center, velvety to floccose, margins regular, pewter at the center and white at the edge, producing a diffusible faint yellow pigment and clear exudates; reverse brown. Aerial mycelium abundant, smooth and thin walled, septate, 1- to 2-μm wide. Terminal and lateral conidia borne on hyphae, short protrusions, or side branches. Conidia solitary, sometimes two in chains, pyriform, sometimes subglobose, smooth or rarely rough walled, ecru, 4.0–6.0 × 3.0–5.5 μm (*av*. = 5.5 × 4.0, *n* = 50). Intercalary conidia abundant, olivary, subglobose to globose, 4.0–7.0 × 3.0–5.5 μm (*av*. = 5.0 × 4.0, *n* = 50).

Sexual morph: not observed.

Geographical distribution: China.

Material examined: China, Guizhou, Guiyang, the Affiliated Hospital of Guizhou Medical University, 26°59′N, 106°71′E, from soil beside a road, September 2016, Zhi. Y. Zhang (HMAS 255397 – holotype; CGMCC 3.18498 = GZUIFR K277.1 – ex-type living cultures; ibid., CGMCC 3.18499 = GZUIFR K277.2; ibid., CGMCC 3.18500 = GZUIFR K277.3). The living cultures were kept in sterile 30% glycerol and deposited in a −80°C freezer.

Notes: *Solomyces sinensis* was isolated from soil in Guizhou Province, China. We did not compare morphological characteristics between *S. sinensis* and another two strains within *Solomyces* owing to the lack of morphological description of these two strains (Minnis and Lindner, 2013). However, *S*. *sinensis* is phylogenetically distinct from these strains with high statistical support (1.00 BYPP, 100% MLBS) (Figures 1, 2).

## Discussion

In this study, *Solomyces* gen. nov. is introduced with an asexual morph. Five new species are also described. All the new taxa belong in the order Thelebolales, the members of which are ubiquitous in the environment. Several taxa belonging to this order have been isolated from tropical to arctic regions. They are often coprophilous and frequently isolated from freshwater and saline lakes (De Hoog et al., 2005). They have also been recorded from soils, epiphytic soils in tree holes (Chen et al., 2017), mine sediments (Crous et al., 2019), and sponges (Bovio et al., 2018). Thelebolales have been recorded as saprobic on dead plant material, rarely as plant-parasitic, and also as animal pathogens, e.g., the well-known white-nose disease of bats (Lorch et al., 2011). In addition, several members of Thelebolales are keratinophilic; i.e., they can invade and degrade keratin material (Saxena et al., 2005). All the proposed new taxa in our study were isolated using the baiting technique, a method specifically designed for the isolation of keratinophilic fungi. Consequently, additional studies are needed to assess whether our new taxa can also degrade keratin material.

Recent studies demonstrated that the Thelebolales comprise at least two families or groups (Ekanayaka et al., 2019; Johnston et al., 2019; Batista et al., 2020). Some studies have proposed that the Thelebolales consists of Pseudeurotiaceae and Thelebolaceae based on three distinct pieces of evidence (568 ITS sequences, 15 genes concatenated from 279 species, and a phylogenomic approach from 51 complete genomes) and on a phylogenomic approach, respectively (Johnston et al., 2019; Batista et al., 2020). The Thelebolaceae, represented by the *Thelebolus* and *Antarctomyces*, share a common ancestor with the family Pseudeurotiaceae, represented by the genus *Pseudogymnoascus* (Johnston et al., 2019; Batista et al., 2020). However, a different study indicated that Pseudeurotiaceae was nested within Thelebolaceae based on phylogenetic analysis and synonymized Pseudeurotiaceae under Thelebolaceae (Ekanayaka et al., 2019). The same authors discovered that several genera, previously classified as Leotiaceae and Leotiomycetes genera *incertae sedis*, clustered within Thelebolales as a sister clade to the Thelebolaceae and defined them as the *Alatospora*–*Miniancora* clade (Ekanayaka et al., 2019). The studies by Ekanayaka et al. contained more genus taxa in Thelebolales; therefore, we continued the phylogenetic analysis in Thelebolales based on this study.

Recent reports have indicated that the Thelebolales contained 22 genera. However, because no ITS and *LSU* sequence data were available for *Ascophanus*, *Ascozonus*, *Caccobius*, *Coprobolus*, *Leptokalpion*, *Neelakesa*, and *Pseudascozonus* (Ekanayaka et al., 2019), we could not compare the phylogenetic relationships between these genera and *Solomyces*. In our phylogenetic analysis, our three isolates (CGMCC 3.18498, CGMCC 3.18499, and CGMCC 3.18500; *Solomyces sinensis*) and two isolates of Minnis and Lindner (2013) (15PA02 and 17WV02, from Hibernacular soil in Pennsylvania and West Virginia, respectively) formed an independent clade with strong statistical support (BYPP 1/MLBS 100%) and were close to *Geomyces*. Morphologically, the asexual stage of *Ascophanus*, *Ascozonus*, *Caccobius*, *Coprobolus*, *Leptokalpion*, *Neelakesa*, and *Pseudascozonus* is not recorded in the literature (Wijayawardene et al., 2017). Therefore, no morphological comparison can be done between these genera and *Solomyces*. However, *Solomyces* differs from Geomyces by terminal and lateral conidia borne on hyphae, short protrusions or side branches, olivary, subglobose to globose intercalary conidia, and absence of the forming verticillate and opposite branches with an acute angle to the axis near the apex of conidiophores (Sigler and Carmichael, 1976).

Based on morphological characteristics, it was difficult to distinguish closely related species, and even genera, using traditional taxonomy, and modern phylogenetic methods were a very important adjunct. Although Crous et al. (2019) described the new species, *P*. *linderi* and *P*. *turneri*, based on the similarity of morphological characteristics between these two new species and *P. bhattii*, they did not compare the phylogenetic relationship. Our phylogenetic analysis indicated that *P. bhattii* (type strain CBS 760.71) was nested within *Gymnostellatospora* (Figure 1), and we, therefore, transferred *P. bhattii* to the *Gymnostellatospora* and named it *G. bhattii*.

## Data Availability Statement

The sequences generated in this study can be found in GenBank. The accession numbers of the sequences deposited in GenBank are ITS: MT509362–MT509372, *LSU*: MT509376–MT509386, *MCM7*: MT534202–MT534212, *RPB2*: MT534216–MT534226, and *EF1A*: MT534227–MT534237.

## Author Contributions

YH and JH were responsible for conceptualization and funding acquisition. ZZ, CD, WC, QM, and XL were responsible for data acquisition. ZZ, CD, and WC did the formal analysis. ZZ wrote the first draft. YH and ZL wrote, reviewed, and edited the manuscript. All authors have read and agreed to the published version of the manuscript.

## Conflict of Interest

The authors declare that the research was conducted in the absence of any commercial or financial relationships that could be construed as a potential conflict of interest.

## Footnotes

1 www.Mycobank.org

2 www.facesoffungi.org

3 http://tree.bio.ed.ac.uk/software/figtree

## References

[B1] Batista T. M. Hilário H. O. de Brito G. A. M. Moreira R. G. Furtado C. de Menezes G. C. A. (2020). Whole-genome sequencing of the endemic Antarctic fungus *Antarctomyces pellizariae* reveals an ice-binding protein, a scarce set of secondary metabolites gene clusters and provides insights on Thelebolales phylogeny. *Genomics* 112 2915–2921. 10.1016/j.ygeno.2020.05.004 32389811

[B2] Bovio E. Garzoli L. Poli A. Prigione V. Firsova D. McCormack G. P. (2018). The culturable mycobiota associated with three Atlantic sponges, including two new species: *Thelebolus balaustiformis* and *T. spongiae*. *Fungal Syst. Evol.* 1 141–167. 10.3114/fuse.2018.01.07 32490365 PMC7259239

[B3] Chen W. H. Zeng G. P. Luo Y. Liang Z. Q. Han Y. F. (2017). Morphological traits and molecular analysis for *Geomyces fujianensis* sp. nov. from China. *Mycosphere* 8 38–43. 10.5943/mycosphere/8/1/5

[B4] Crous P. W. Wingfiled M. J. Lombard L. Roets F. Swart W. J. Alvarado P. (2019). Fungal planet description sheets: 951–1041. *Persoonia* 43 223–425. 10.3767/persoonia.2019.43.06 32214501 PMC7085856

[B5] Dal Vesco G. (1957). *Geomyces vinaceus*” n. sp. forma conidica di “*Pseudogymnoascus vinaceus*. *Raillo. Allionia* 3 1–15.

[B6] De Hoog G. S. Göttlich E. Platas G. Genilloud O. Leotta G. van Brummelen J. (2005). Evolution, taxonomy and ecology of the genus *Thelebolus* in Antarctica. *Stud. Mycol.* 51 33–76.

[B7] De Menezes G. C. Godinho V. M. Porto B. A. Gonçalves V. N. Rosa L. H. (2017). *Antarctomyces pellizariae* sp. nov., a new, endemic, blue, snow resident psychrophilic ascomycete fungus from Antarctica. *Extremophiles* 21 259–269. 10.1007/s00792-016-0895-x 27900476

[B8] Drummond A. Rambaut A. (2007). BEAST: bayesian evolutionary analysis by sampling trees. *BMC Evol. Biol.* 7:214. 10.1186/1471-2148-7-214 17996036 PMC2247476

[B9] Eckblad F. E. (1968). The genera of the operculate discomycetes. A re-evaluation of their taxonomy, phylogeny and nomenclature. *Norw. J. Bot.* 15 1–191.

[B10] Ekanayaka A. Hyde K. D. Gentekaki E. McKenzie E. H. C. Zhao Q. Bulgakov T. S. (2019). Preliminary classification of Leotiomycetes. *Mycosphere* 10 310–489. 10.5943/mycosphere/10/1/7 26907596

[B11] Ekanayaka A. H. Ariyawansa H. A. Hyde K. D. Jones E. B. G. Daranagama D. A. Phillips A. J. L. (2017). Discomycetes: the apothecial representatives of the phylum Ascomycota. *Fungal Divers.* 87 237–298. 10.1007/s13225-017-0389-x

[B12] Eriksson O. E. Winka K. (1997). Supraordinal taxa of Ascomycota. *Myconet.* 1 1–16.

[B13] Gargas A. Trest M. T. Christensen M. Volk T. J. Blehert D. S. (2009) Geomyces destructans sp. nov. associated with bat white-nose syndrome. *Mycotaxon* 108, 147–154. 10.5248/108.147

[B14] Haeckel E. (1894). *Systematische Phylogenie. Entwurf eines Natürlichen Systems der Organismen auf Grund ihrer Stammesgeschichte. Theil1, Systematische Phylogenie der Protisten und Pflanzen.* (Berlin: G. Reimer), 1–400.

[B15] Hocking A. D. Pitt J. I. (1988). Two new species of xerophilic fungi and a further record of *Eurotium halophilicum*. *Mycologia* 80 82–88. 10.2307/3807497

[B16] Johnston P. R. Quijada L. Smith C. A. Baral H.-O. Hosoya T. Baschien C. (2019). A multigene phylogeny toward a new phylogenetic classification of Leotiomycetes. *IMA Fungus* 10:1. 10.1186/s43008-019-0002-x 32647610 PMC7325659

[B17] Kalyaanamoorthy S. Minh B. Q. Wong T. K. von Haeseler A. Jermiin L. S. (2017). ModelFinder: fast model selection for accurate phylogenetic estimates. *Nat. Methods* 14 587–589. 10.1038/nmeth.4285 28481363 PMC5453245

[B18] Karunarathna A. Peršoh D. Ekanayaka A. H. Jayawardena R. S. Thilini Chethana K. W. Goonasekara I. D. (2020). *Patellariopsidaceae* Fam. Nov. With Sexual-Asexual Connection and a New Host Record for Cheirospora botryospora (Vibrisseaceae, Ascomycota). *Front. Microbiol.* 11:906. 10.3389/fmicb.2020.00906 32528427 PMC7264944

[B19] Katoh K. Standley D. M. (2013). MAFFT multiple sequence alignment software version 7: improvements in performance and usability. *Mol. Biol. Evol.* 30 772–780. 10.1093/molbev/mst010 23329690 PMC3603318

[B20] Korf R. P. (1973). “Discomycetes and Tuberales,” in *The Fungi: An Advanced Treatise*, Vol. IVA, eds Ainsworth G. C. Sparrow F. K. Sussman A. S. (New York, NY: Academic Press).

[B21] Li Z. Q. Cui C. Q. (1989). Study on the Psychrophilic/Psychrotrophic microorganisms. III. Geomyces laevis, a new species of Geomyces. *Mycosystema* 8 47–50.

[B22] Liu Y. J. Whelen S. Hall B. D. (1999). Phylogenetic relationships among Ascomycetes: evidence from an RNA polymerase II subunit. *Mol. Biol. Evol.* 16 1799–1808. 10.1093/oxfordjournals.molbev.a026092 10605121

[B23] Lorch J. M. Meteyer C. U. Behr M. J. Boyles J. G. Cryan P. M. Hicks A. C. (2011). Experimental infection of bats with Geomyces destructans causes white-nose syndrome. *Nature* 480 376–378. 10.1038/nature10590 22031324

[B24] Luo Y. Chen W. H. Wang Y. Han Y. F. Liang Z. Q. (2016). A new *Geomyces* species producing melanin in the medium. *Mycosystema* 35 123–130. 10.13346/j.mycosystema.140246

[B25] Minh Q. Nguyen M. von Haeseler A. A. (2013). Ultrafast approximation for phylogenetic bootstrap. *Mol. Biol. Evol.* 30 1188–1195. 10.1093/molbev/mst024 23418397 PMC3670741

[B26] Minnis A. M. Lindner D. L. (2013). Phylogenetic evaluation of *Geomyces* and allies reveals no close relatives of *Pseudogymnoascus destructans*, comb. nov., in bat hibernacula of eastern North America. *Fungal Biol.* 117 638–649. 10.1016/j.funbio.2013.07.001 24012303

[B27] Nguyen L. T. Schmidt H. A. von Haeseler A. Minh B. Q. (2015). IQ-TREE: a fast and effective stochastic algorithm for estimating maximum-likelihood phylogenies. *Mol. Biol. Evol.* 32 268–274. 10.1093/molbev/msu300 25371430 PMC4271533

[B28] Posada D. Crandall K. A. (1998). Modeltest: testing the model of DNA substitution. *Bioinformatics* 14 817–818. 10.1093/bioinformatics/14.9.817 9918953

[B29] Reeb V. Lutzoni F. Roux C. (2004). Contribution of RPB2 to multilocus phylogenetic studies of the euascomycetes (Pezizomycotina, Fungi) with special emphasis on the lichen-forming Acarosporaceae and evolution of polyspory. *Mol. Phylogenet. Evol.* 32 1036–1060. 10.1016/j.ympev.2004.04.012 15288074

[B30] Rehner S. A. Buckley E. (2005). A *Beauveria* phylogeny inferred from nuclear ITS and EF1-alpha sequences: evidence for cryptic diversification and links to *Cordyceps teleomorphs*. *Mycologia* 97 84–98. 10.3852/mycologia.97.1.84 16389960

[B31] Rice A. V. Currah R. S. (2006). Two new species of *Pseudogymnoascus* with *Geomyces* anamorphs and their phylogenetic relationship with *Gymnostellatospora*. *Mycologia* 98 307–318. 10.3852/mycologia.98.2.307 16894976

[B32] Ronquist F. Teslenko M. van der Mark P. Ayres D. L. Darling A. Höhna S. (2012). MrBayes 3.2: efficient Bayesian phylogenetic inference and model choice across a large model space. *Syst. Biol.* 61 539–542. 10.1093/sysbio/sys029 22357727 PMC3329765

[B33] Samson R. A. (1972). Notes on *Pseudogymnoascus*, *Gymnoascus* and related genera. *Acta Bot. Neerlandic.* 21 517–527. 10.1111/j.1438-8677.1972.tb00804.x

[B34] Sati S. C. Pathak R. (2016). Anamorph (asexual stage) Teleomorph (sexual stage) Connections in Aquatic hyphomycetes. *Int. J. Plant Reprod. Biol.* 8 128–135.

[B35] Saxena P. Kumar A. Shrivastava J. N. (2005). Keratinophilic fungi: a microbial way to manage poultry waste feathers. *Indian J. Microbiol.* 45 151–154.

[B36] Schoch C. L. Sung G. H. López-Giráldez F. Townsend J. P. Miadlikowska J. Hofstetter V. (2009). The Ascomycota Tree of Life: a phylum-wide phylogeny clarifies the origin and evolution of fundamental reproductive and ecological traits. *Syst Biol.* 58 224–239. 10.1093/sysbio/syp020 20525580

[B37] Sigler L. Carmichael J. W. (1976). Taxonomy of *Malbranchea* and some other hyphomycetes with arthroconidia. *Mycotaxon* 4 349–488.

[B38] Spooner B. M. (1987). Helotiales of Australasia: geoglossaceae, Orbiliaceae, Scelrotiniaceae, Hyaloscyphaceae. *Bibl. Mycol.* 116 1–711.

[B39] Stchigel A. M. Josep C. A. Mac Cormack W. Guarro J. (2001). *Antarctomyces psychrotrophicus* gen. et sp. nov., a new ascomycete from Antarctica. *Mycol. Res.* 105 377–382. 10.1017/s0953756201003379

[B40] Sutton B. C. Hennebert G. L. (1995). “Interconnections amongst anamorphs and their possible contribution to ascomycetes systematics,” in *Ascomycete Systematics: Problems and Perspectives in the Nineties*, ed. Hawksworth D. L. (New York, NY: Plenum), 77–100.

[B41] Swofford D. L. (2002). *PAUP^∗^ 4.0b10: Phylogenetic Analysis Using Parsimony (^∗^and other methods).* Sunderland, MA: Sinauer.

[B42] Tamura K. Stecher G. Peterson D. Filipski A. Kumar S. (2013). MEGA6: molecular evolutionary genetics analysis version 6.0. *Mol. Biol. Evol.* 30 2725–2729. 10.1093/molbev/mst197 24132122 PMC3840312

[B43] Traaen A. E. (1914). Untersuchungen über bodenpilze aus Norwegen. *Nytt Mag. Naturvidensk.* 52 19–121.

[B44] Vaidya G. Lohman D. J. Meier R. (2011). SequenceMatrix: concatenation software for the fast assembly of multi-gene datasets with character set and codon information. *Cladistics* 27 171–180. 10.1111/j.1096-0031.2010.00329.x 34875773

[B45] Van Brummelen J. (1985). *Pseudoascozonus*, a new genus of Pezizales. *Proc. Indian Acad. Sci.* 94 363–367.

[B46] Van Oorschot C. A. (1980). A revision of chrysosporium and allied genera (No. 20). *Stud. Mycol.* 20 1–89.

[B47] Vanbreuseghem R. (1952). Technique biologique pour l’isolement des dermatophytes du sol. *Ann. Soc. Belg. Med. Trop.* 32 175–178. 14953089

[B48] Vilgalys R. Hester M. (1990). Rapid genetic identification and mapping of enzymatically amplified ribosomal DNA from several *Cryptococcus* species. *J. Bacteriol.* 172 4238–4246. 10.1128/jb.172.8.4238-4246.1990 2376561 PMC213247

[B49] Vilgalys R. Sun B. L. (1999). Ancient and recent patterns of geographic speciation in the oyster mushroom *Pleurotus* revealed by phylogenetic analysis of ribosomal DNA sequences. *Proc. Natl. Acad. Sci. U.S.A.* 91 4599–4603. 10.1073/pnas.91.10.4599 8183955 PMC43833

[B50] White T. J. Bruns T. Lee S. Taylor J. (1990). “Amplification and direct sequencing of fungal ribosomal RNA genes for phylogenetics,” in *PCR Protocols*, eds Innis M. A. Gelfand D. H. Sninsky J. J. White T. J. (San Diego, CA: Academic Press), 315–322. 10.1016/B978-0-12-372180-8.50042-1

[B51] Wijayawardene N. N. Hyde K. D. Rajeshkumar K. C. Hawksworth D. L. Madrid H. Kirk P. M. (2017). Notes for genera: Ascomycota. *Fungal Divers.* 86 1–594. 10.1007/s13225-017-0386-0

[B52] Zhang Z. Y. Chen W. H. Zou X. Han Y. F. Huang J. Z. Deshmukh S. K. (2019). Phylogeny and taxonomy of two new *Plectosphaerella* (Plectosphaerellaceae, Glomerellales) species from China. *MycoKeys* 57 47–60. 10.3897/mycokeys.57.36628 31423085 PMC6694076

